# Performance of A Priori and A Posteriori Calibration Strategies in Divergence Time Estimation

**DOI:** 10.1093/gbe/evaa105

**Published:** 2020-05-22

**Authors:** Alan J S Beavan, Philip C J Donoghue, Mark A Beaumont, Davide Pisani

**Affiliations:** e1 School of Biological Sciences, University of Bristol, United Kingdom; e2 School of Earth Sciences, University of Bristol, United Kingdom

**Keywords:** molecular clock, Bayesian, RelTime, simulation

## Abstract

Relaxed molecular clock methods allow the use of genomic data to estimate divergence times across the tree of life. This is most commonly achieved in Bayesian analyses where the molecular clock is calibrated a priori through the integration of fossil information. Alternatively, fossil calibrations can be used a posteriori, to transform previously estimated relative divergence times that were inferred without considering fossil information, into absolute divergence times. However, as branch length is the product of the rate of evolution and the duration in time of the considered branch, the extent to which a posteriori calibrated, relative divergence time methods can disambiguate time and rate, is unclear. Here, we use forward evolutionary simulations and compare a priori and a posteriori calibration strategies using different molecular clock methods and models. Specifically, we compare three Bayesian methods, the strict clock, uncorrelated clock and autocorrelated clock, and the non-Bayesian algorithm implemented in RelTime. We simulate phylogenies with multiple, independent substitution rate changes and show that correct timescales cannot be inferred without the use of calibrations. Under our simulation conditions, a posteriori calibration strategies almost invariably inferred incorrect rate changes and divergence times. The a priori integration of fossil calibrations is fundamental in these cases to improve the accuracy of the estimated divergence times. Relative divergence times and absolute timescales derived by calibrating relative timescales to geological time a posteriori appear to be less reliable than a priori calibrated, timescales.

## Introduction

Evolutionary biologists have long sought the timescale of life (Simpson 1944; Betts et al. 2018). Although traditionally this was done using fossils, the molecular clock (Zuckerkandl and Pauling 1965a, 1965b) has emerged as a powerful tool for extending the value of the fossil record to calibrating molecular evolution to geologic time and, thereby approaching an accurate timescale for the history of life (Avise et al. 1992; Erwin et al. 2011; Claramunt and Cracraft 2015; Dos Reis et al. 2015; Betts et al. 2018; Morris et al. 2018). Early molecular clock applications relied on an assumption of near constant evolutionary rate across the sequences being studied (see Runnegar 1982; Wray et al. 1996). However, the substitution rate evolves according to the same principles by which other traits evolve (Bromham 2009), resulting in stochastic changes that can be difficult to model (Kimura 1983; Ohta 1992). Evolution of the substitution rate is influenced by a combination of factors including mutation rate, generation time, and population genetic factors such as the strength of selection for a given allele and the effective population size (Lynch 2010; Lanfear et al. 2014; Lehtonen and Lanfear 2014). Indeed, extensive rate variation across sites and lineages is broadly documented and rate constancy is rare across distantly related taxa (Langley and Fitch 1974; Baldwin et al. 1995; Lopez et al. 2002; Smith and Donoghue 2008; Bromham 2009; Duchene et al. 2016). The new millennium ushered in a diversity of “relaxed” molecular clock models (Sanderson 1997; Thorne et al. 1998; Drummond et al. 2006), motivated by these findings, promising better estimation of divergence times when evolutionary rates are variable.

Molecular clocks can be calibrated by fossils, known substitution rate, known sampling dates, or timing of historical biogeographic events (Hipsley and Müller 2014). Molecular clock methods differ in the way they treat these calibrations. Rate and time are confounded as recently reiterated also by dos Reis and Yang (2013) and Zhu et al. (2015) in estimates of branch length, as the length of a branch is the product of the rate of substitution and the length of the branch in time. This implies that any given branch length (expressed in substitutions per site) can be the result of infinite combinations of branch length (in time) and substitution rate per site per unit of time. Accordingly, it has long been customary to integrate a priori at the least one calibration to disambiguate rates and times along the branches of a tree. Where evolution is clock-like only one calibration is necessary, but where the clock assumption is violated, more calibrations are required to disambiguate time and rate across the tree. However, the a priori integration of fossil calibrations is not strictly necessary to transform branch lengths into divergence times. In Bayesian and Maximum Likelihood frameworks, relative rates of substitution and relative divergence times can be calculated, based on molecular data only, by arbitrarily assigning the age of the root node (e.g., to 1, 100, 1,000, 1,000,000, or any other values) to derive a relative timescale. Although these relative timescales cannot, on their own, help us infer the absolute age of nodes in a tree, they can be used to make inferences on the age of nodes in a tree relative to each other (e.g., Loader et al. 2007). In addition, these relative timescales can be calibrated a posteriori to generate absolute divergence times. This strategy should succeed when molecular evolution is clock-like, but without multiple calibrations or a more sophisticated algorithm of branch length comparison, substitution rates cannot be estimated accurately for sequences that have not evolved under a strict clock, when the root node only is calibrated. As pointed out above, this is because every branch length (in substitution per sites) in phylogenies derived from such data can be explained by an infinite number of combinations of rates and times. Without disambiguating rates and times (using multiple calibrations), molecular clock algorithms cannot be expected to have the power to accurately estimate every location and magnitude of rate change across a phylogeny from the sequence data only, regardless of the length of the alignment (Britton 2005).

An alternative approach to estimating relative rates of evolution is to infer relative rates of sister lineages, under the assumption that all lineages descending from the same node have been evolving for the same amount of time. This approach, implemented in the software RelTime (Tamura et al. 2012), assigns relative rate ratios progressively from tips to root. As in the case of Bayesian relative timescales, RelTime-inferred relative timescales can be calibrated a posteriori to infer absolute divergence times (e.g., Marin et al 2017). The RelTime algorithm cannot incorporate calibrations a priori. Tamura et al. (2012) (see also Mello et al. 2017; Battistuzzi et al. 2018) suggested that the a posteriori transformation of relative divergence times into absolute divergence times in RelTime is to be preferred to the a priori integration of fossils in divergence time estimation because it shields the method from the potentially negative effects associated with the inclusion of incorrect calibrations on the estimation of rates of evolution. According to these authors, the principal weakness of standard divergence time estimates is that they are vulnerable to inaccurate interpretations of fossil evidence used in formulating calibrations, especially with reference to the placement of upper bounds on the ages of clades and the construction of a joint time prior from individual clade age priors. This, in their view, can constrain molecular signal and lead to the recovery of erroneous divergence time estimates. Whether deriving a relative timescale (without fossil calibrations) using the RelTime tip-to-root process can accurately infer substitution rates and, by extension, divergence times, is not clear and needs to be tested against Bayesian alternatives using fossil calibrations.

Here, we explore the performance of a priori and a posteriori approaches to calibrating molecular clock methods under the challenging conditions of multiple independent substitution rate changes correlated in time. We ask if calibrations implemented a priori and a posteriori can accurately disambiguate rates and times under these simulated conditions. We compare the application of a strict, uncorrelated, and autocorrelated clock, implemented in a Bayesian setting, and the RelTime algorithm which can be defined as a local clock or uncorrelated relaxed clock method (Ho and Duchene 2014). Different sets of calibrations were implemented, the effects of which were compared across each method. We found that the use of relative timescales and the a posteriori calibration of relative timescales systematically fails to correctly estimate rate variation and divergence times. These results suggest that, irrespective of the implementation, relaxed relative divergence time approaches are not reliable for dating the tree of life—unless we can be confident that the data evolved under a clock, or we can rule out the possibility that multiple, independent substitution rate changes affect the data.

## Results

In all cases presented here, the sequences were not simulated under a model that is implemented in the software that we evaluate. Rather, the sequences were simulated under a scenario explicitly designed to be difficult for model-based approaches to resolve. In all cases, the tree topology was fixed for both simulation and inference. In all figures, the distributions represent the mean posterior ages estimated using Bayesian methods or the ages inferred by RelTime across all simulations.

### Changes in Evolutionary Rate Correlated in Time across the Phylogeny Cannot Be Correctly Detected without Calibrations

**Fig. 1.**
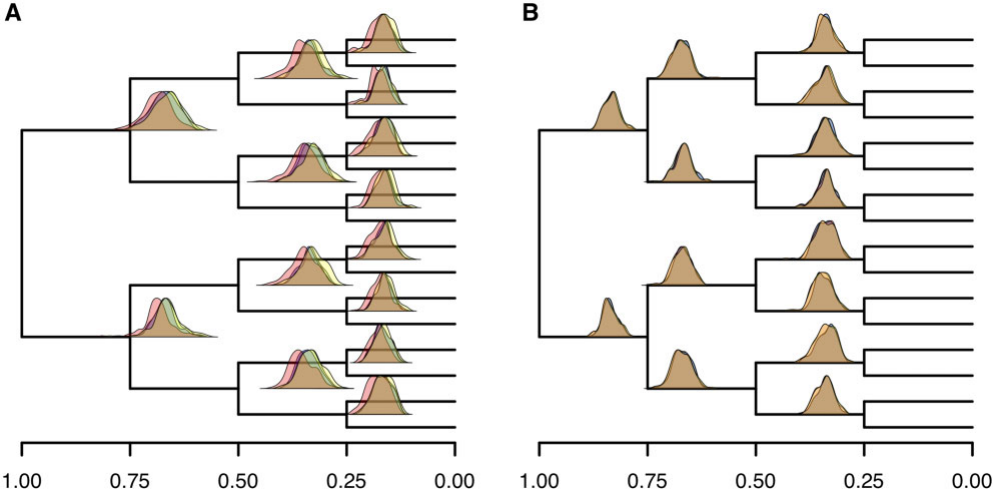
Without calibration, all methods estimate branch times directly proportional to the branch lengths. Distributions of mean divergence time estimates for strict clock (green), uncorrelated clock (blue), autocorrelated clock (red), and RelTime (yellow) are shown for each node whose age is estimated, in 100 replicates. The location of the node on the *y* axis corresponds to a frequency of 0 on the *y* axis of the distribution for that node. Where distributions overlap, the colors merge. (*A*) Fifty percent reduction in mutation rate at 50,000 generations dated relatively and (*B*) 100% increase in the mutation rate at 50,000 generations dated relatively.

We simulated 100 replicates of evolution of populations of individuals on a 17 species tree (with a symmetrical topology of 16 ingroup taxa and 1 outgroup taxon). Speciation events were simulated along the 17 species tree after each branch had been evolving (independently) for 25,000 generations, with the exception of the outgroup. In this way, the root of the tree was 100,000 generations before the present (represented by the 16 ingroup taxa). In the first set of simulations, either a doubling or a halving of the substitution rate was assumed to have occurred concomitantly and identically across all ancestors, 50,000 generations before present (supplementary fig. S1*A* and *B*, Supplementary Material online). We first estimated relative divergence times applying the RelTime local clock algorithm and using a standard Bayesian method where the root node was set to have an arbitrary age of 1 (e.g., Lozano-Fernandez et al. 2017). Bayesian analyses were performed using strict, lognormal-autocorrelated, and uncorrelated gamma clock models. Under these experimental conditions, all molecular clock methods and clock models are expected to fail, and this analysis was performed as a control. This is because there is no signal of the rate change in an ultrametric, symmetrical tree with independent, identical substitution rate changes at the same point in time (see Ho et al. 2011). As expected, all models and methods failed to estimate the correct divergence times under these conditions (fig. 1). In all our results, the estimated mean branch times were directly proportional to branch length, meaning branches evolving at higher rates were inferred to be longer in time than slow branches. Furthermore, the simulation generating ages were not captured by the 95% confidence intervals in RelTime, or the 95% credibility intervals for the Bayesian analyses, although some node ages did fall at the extreme ends of the confidence intervals in a small subset of the 100 simulated data sets. Whether the rate increased or decreased at 50,000 generations before present did not make any difference in the overall accuracy of results, as results obtained when rates increased or decreased were symmetrical. Accordingly, we only used the set of simulations where the substitution rate decreased at 50,000 generations in the past across all lineages (fig. 1*A*) in subsequent analyses performed to evaluate the effect of including calibrations when the rate change was identical in all lineages (see below). We are satisfied that the findings would be mirrored if we had simulated rate increases at 50,000 instead of decreases, provided the magnitude of change was identical.

### When Rates Change Simultaneously in Independent Lineages the Calibration Strategy Used Predicts Whether Correct Divergence Times Can Be Estimated

Calibrations were applied to the same data sets used in analyses that did not use calibrations (see above). Different a priori calibration strategies were compared with similar strategies implemented a posteriori in RelTime. In the Bayesian analyses, using either a strict, an uncorrelated or an autocorrelated clock, a priori calibrations were defined by uniform prior distributions on divergence times with hard bounds 1,000 generations before and after the generating age of the calibrated node. In RelTime analyses, calibrations constrained the ages of the nodes to within 1,000 generations of their generating age.

**Fig. 2.**
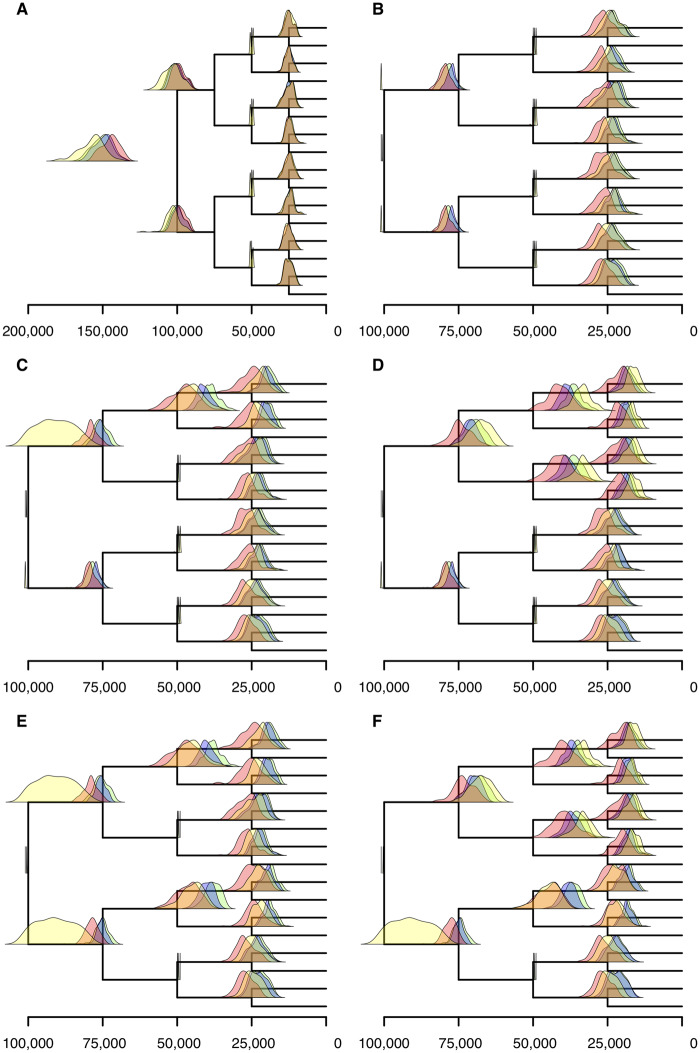
Bayesian estimation of divergence times improves with calibrations of nodes corresponding to a rate change. Distributions of mean divergence time estimations using strict clock (green), uncorrelated clock (blue), autocorrelated clock (red), and RelTime (yellow) are shown for a tree where all the populations experience a reduction of rate at 50,000 generations, for 100 simulations. Calibrations were placed on all nodes at 50,000 generations in (*A*). In (*B*–*F*), the root was calibrated with additional calibrations at (*B*) all nodes at 50,000 generations, (*C*) three nodes at 50,000 generations, (*D*) two nodes at 50,000 generations in the same eight-taxon subtree, (*E*) two nodes at 50,000 generations in different eight-taxon subtrees, and (*F*) one node at 50,000 generations. Where the distribution for the age of one node extends beyond the parent (e.g., G), this is a smoothing artifact in the presentation of the distributions. No inferred ages of nodes are older than their parents.

First, Bayesian analyses were performed applying a priori calibrations to all nodes where the rate changed (fig. 2*A* and supplementary fig. S1*C*, Supplementary Material online), but without calibrating the root node. Absolute divergence times were obtained from the RelTime-estimated relative timescale calibrating the same nodes as in the Bayesian analyses, but a posteriori. Analyses were then repeated adding also a calibration to the root node (fig. 2*B* and supplementary fig. S1*D*, Supplementary Material online). Subsequently, increasingly relaxed calibration strategies were tested for both the Bayesian analyses and RelTime. In these analyses, the root was always calibrated and calibrations were removed from the four internal nodes, first removing one internal calibration (fig. 2*C* and supplementary fig. S1*E*, Supplementary Material online), then two calibrations. Here, two conditions were tested: In the first, the two calibrations that were removed were located in the same eight-taxon subtree (fig. 2*D* and supplementary fig. S1*F*, Supplementary Material online). In the second, they were on the different subtrees (fig. 2*E* and supplementary fig. S1*G*, Supplementary Material online). Finally, three calibrations were removed, leaving only one internal, calibrated node (fig. 2*F* and supplementary fig. S1*H*, Supplementary Material online).

All Bayesian analyses where the root node was not calibrated failed to estimate correct divergence times, overestimating the duration of branches with higher substitution rates (fig. 2*A*), exactly as in the case when relative divergence times were calculated (fig. 1). That is, the correct ages of nodes at 75,000 and 100,000 generations were never within the 95% credibility intervals of estimated node ages. For the RelTime analyses, the same set of calibrations was enforced but a posteriori. Exactly, as in the case of the Bayesian analyses, the generating node age never fell within the 95% confidence intervals for these nodes.

When five nodes were calibrated (all nodes corresponding to a rate change and the root), all Bayesian analyses recovered accurate divergence time estimates with a slight overestimation of the age of the nodes at 75,000 generations under the strict and autocorrelated clock models (fig. 2*B*). For these nodes, the generating node age was contained within the 95% credibility intervals in 54%, 100%, and 55% of cases for the strict, uncorrelated, and autocorrelated clock models, respectively. These results clearly illustrate the importance of incorporating a calibration for the root node in Bayesian divergence time analyses in the absence of a strict molecular clock. For nodes at 25,000 generations in the past, the true age of the node placed within the 95% credibility intervals 93.5%, 97.8%, and 95.4% of times in the strict clock, uncorrelated clock, and autocorrelated clock analyses, respectively. Similarly, for these nodes, the a posteriori calibrated RelTime algorithm accurately estimated divergence times in 81.6% of analyses. However, for deep nodes in the tree, whose generating age was 75,000 generations into the past, RelTime continued to overestimate node ages, systematically failing to detect a rate variation 50,000 generations in the past, and estimating branch times equal to 0 in many cases (e.g., fig. 2*B*, nodes at 75,000 generations). Specifically, RelTime gave confidence intervals containing the generating node age in only 3% of replicates. These cases were highly unusual. In all other replicated analyses, RelTime placed the lower bound of the confidence interval for nodes generated at 75,000 generations at more than 90,000 generations in the past. Even in the accurate cases, the mean node age estimates for these nodes was at 98,000 generations or above. Why RelTime infers branch times of 0 is unclear. We investigated the output files to find what rate was being inferred here, as it should tend to infinity as the branch length approaches 0. Differently, we found that in all cases where the inferred branch time was 0, the inferred rate was exactly 1.

When internal calibrations were removed, irrespective of how they were distributed, we found that the age estimates for nodes subtending the retained calibrations (true node age 25,000 generations) were mostly accurate (75.1%, 99.9%, 95.8%, and 97.3% in analyses using strict clock, uncorrelated clock, autocorrelated clock, and RelTime, respectively, fig. 2). This shows age estimates were not greatly affected by the removal of calibrations on nodes that were not direct parents of the node in question. When the parent node was not calibrated for these nodes, the accuracy was 14.2%, 88.9%, 96.1%, and 73.8%, respectively, for the strict clock (fig. 2), uncorrelated clock, autocorrelated clock, and RelTime. For nodes at 75,000 generations, the accuracy of divergence time estimation depended on the calibration strategy. The parent (root) node was always calibrated. When both daughters were calibrated (fig. 2*B*–*D*) accuracy was 59.5%, 99.5%, 59.5%, and 50%, when only one was calibrated (fig. 2*C*, *E*, and *F*) it was 92%, 100%, 77%, and 95.3%, and when neither was calibrated (fig. 2*D*–*F*) it was 46%, 100%, 99.5%, and 61.5% for the strict clock, uncorrelated clock, autocorrelated clock, and RelTime, respectively. In RelTime, the age estimates for these nodes are pushed back to the root but the 95% confidence interval often extended beyond the true node age of 75,000 generations; thus, the estimates are accurate as a consequence of their substantial imprecision. The RelTime mean ages for these nodes had a very diffuse distribution when only one daughter node was calibrated (fig. 2*C*, *E*, and *F*).

In noncalibrated parts of the tree (eight-taxon subtrees without calibrations), strict clocks and uncorrelated clocks tended to underestimate node ages leading to inference of erroneously fast evolving branches emerging from these nodes and slowly evolving branches leading to them, with the autocorrelated model achieving greatest accuracy (generating node ages were encompassed by the 95% credibility interval in 46%, 100%, 99.5%, and 61.5% of cases for nodes at 75,000 generations, 0%, 44.8%, 77.3%, and 27.8% for nodes at 50,000 generations, and 8.8%, 83.5%, 93.6%, and 48.3% for nodes at 25,000 generations, for strict clock, uncorrelated clock, autocorrelated clock, and RelTime, respectively). The presence or absence of calibrations in the opposing eight-taxon subtree made little difference to the accuracy and precision of the node age estimates on uncalibrated subtrees in Bayesian analyses. However, for RelTime, despite the mean ages being almost identical, the confidence intervals were narrower if more nodes were calibrated in the rest of tree, resulting in fewer of the analyses producing age ranges that include the generating node age. Generally, estimates of how much rates vary across branches (sigma2 parameter in PhyloBayes) increased with the number of calibrations in the Bayesian relaxed clock analyses.

### No Method Can Estimate Divergence Times Reliably without Calibrations When Substitution Rate Independently Varies in Different Directions across the Tree

**Fig. 3.**
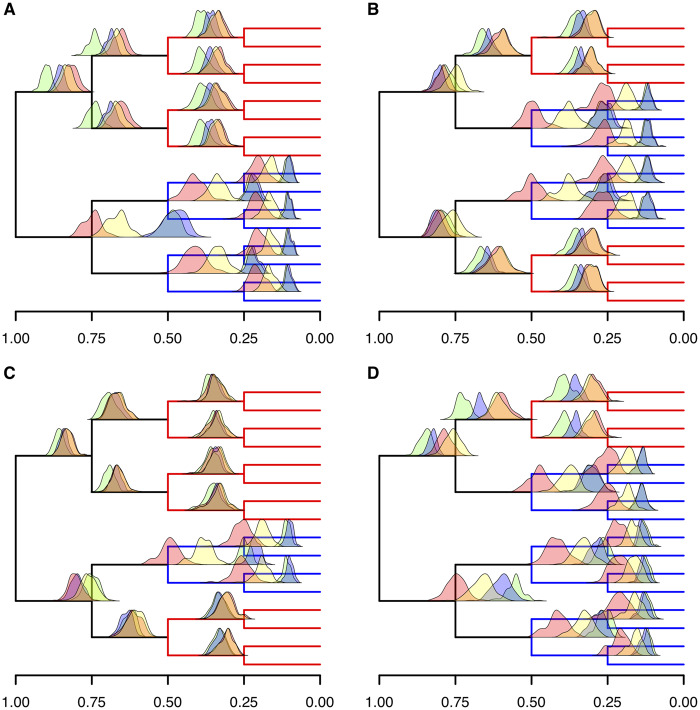
Without calibration, the autocorrelated clock and RelTime detect rate changes but underestimate their magnitude when substitution rate increases and decreases in the same tree. Distributions of mean relative divergence time estimations using strict clock (green), uncorrelated clock (blue), autocorrelated clock (red), and RelTime (yellow) from 100 repeated simulations. In all, fast evolving branches are shown in red and slow evolving branches in blue. The rate changes are characterized as (*A*) 100% increase in rate at 50,000 generations at nodes in the same eight-taxon subtree and 50% reduction at the remaining nodes at 50,000 generations, (*B*) 50% reduction in rate at two nodes at 50,000 at nodes on opposite eight-taxon subtrees and 100% increase at the other nodes at 50,000 generations, (*C*) 100% increase in three nodes at 50,000 generations and 50% reduction at the other, and (*D*) 50% reduction in rate at three nodes at 50,000 generations and 100% increase at the other.

We performed simulations where the rate of evolution changed in different directions (increased at some nodes and decreased at others) along the tree. In all simulations, nodes at 50,000 generations experienced either a 100% increase or a 50% decrease in rate. The rate remained constant elsewhere in the tree. In the first set of simulations, the rate increased in one of the two eight-taxon subtrees and decreased in the other (fig. 3*A* and supplementary fig. S1*I*, Supplementary Material online). We then performed simulations where 1) one rate increase and one rate decrease were simulated in each eight-taxon subtree (fig. 3*B* and supplementary fig. S1*J*, Supplementary Material online) and 2) three rate increases and one decrease (fig. 3*C* and supplementary fig. S1*K*, Supplementary Material online) or 3) three rate decreases and one increase (fig. 3*D* and supplementary fig. S1*L*, Supplementary Material online). Bayesian and RelTime analyses were then performed with no calibrations, producing relative timescales that we scaled so that the root age was fixed at 1. In all cases, the strict and uncorrelated clock models produced similarly overestimated divergence times when leading to branches of high evolutionary rate, and similarly underestimated divergence times when leading to slow evolving lineages. The ability of the uncorrelated rates model to allow for rates to vary among lineages resulted in larger credibility intervals and greater accuracy for this model, with the 95% credibility interval of individual analyses including the correct divergence time in 57.8% of the nodes (excluding the root) across all analyses. By contrast, the strict clock model was accurate in only 6.1% of nodes across all analyses.

Under these conditions, both RelTime and the autocorrelated clock model correctly inferred the direction of rate changes, producing more accurate divergence times than the strict and the uncorrelated clocks. The autocorrelated clock was most accurate when inferring node ages within slowly evolving clades, where it did not underestimate the change in rate. For all other nodes, the magnitude of rate change was underestimated by both the autocorrelated clock model and the local clock algorithm of RelTime, with RelTime predicting mean ages further from the generated node ages in most cases (fig. 3). However, the confidence intervals for RelTime tend to be broader than the credibility intervals for the node ages estimated by the Bayesian autocorrelated clock, thus achieving greater accuracy through greater imprecision (96.7% for RelTime compared with 85.1% for the autocorrelated clock).

### A Priori Calibration Strategies Improve Divergence Time Estimation When Rate Changes in Different Directions in the Tree

**Fig. 4.**
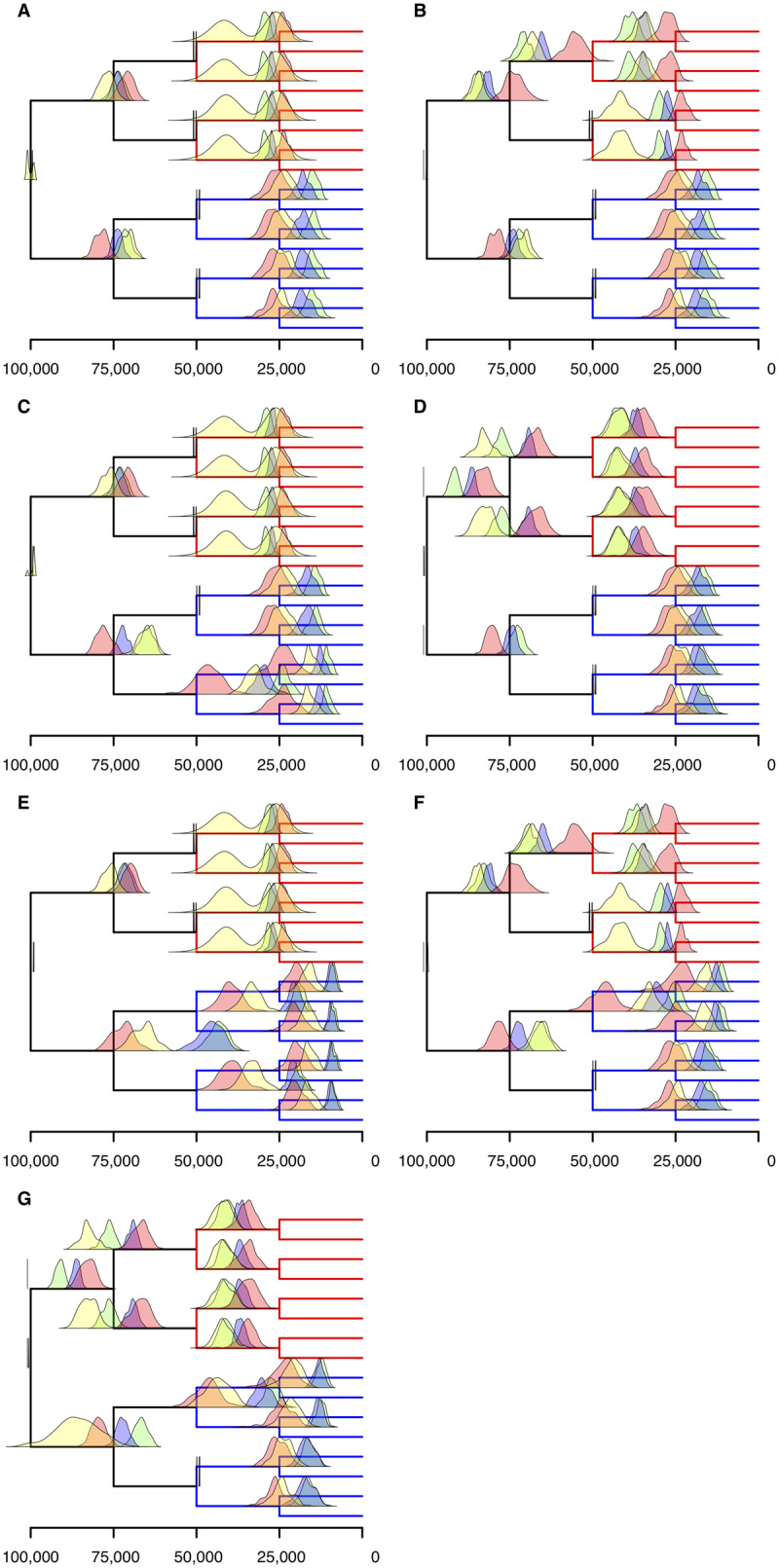
Bayesian estimation of divergence times improves with calibration of nodes corresponding to a rate change when substitution rate increases and decreases in the same tree. Distributions of mean divergence time estimations using strict clock (green), uncorrelated clock (blue), autocorrelated clock (red), and RelTime (yellow) for 100 repeated simulations. In all, the root is calibrated in addition to (*A*) all nodes at 50,00 generations, (*B*) both nodes with a reduction in rate and one with an increase, (*C*) both nodes with an increase in rate and one with a reduction, (*D*) both nodes with a reduction in rate, (*E*) both nodes with an increase in rate, (*F*) one node with an increase in rate and one with a decrease, and (*G*) one node with a reduction in rate. Where the distribution for the age of one node extends beyond the parent (e.g., G), this is a smoothing artifact in the presentation of the distributions. No inferred ages of nodes are older than their parents.

We quantified the extent to which calibrations improve accuracy of divergence time estimation with reference to one of the cases considered above. We considered the case where two independent rate increases were applied to one subtree and two decreases were applied to the other subtree (supplementary fig. S1*I*, Supplementary Material online) as this was the scenario with the lowest accuracy across all our molecular clock analyses. As previously, a priori calibrations were applied to Bayesian analyses with hard bounds 1,000 generations before and after the true generating age of the calibrated node. In RelTime, the age of calibrated nodes was restrained to be within 1,000 generations of their generating age. For these tests, the root was calibrated and further calibrations were placed on nodes at 50,000 generations. First, we calibrated all four nodes (fig. 4*A* and supplementary fig. S1*M*, Supplementary Material online). Subsequently, six experiments were performed where subsets of calibrations were removed. We removed 1) a calibration placed on a node subtending a rate increase (fig. 4*B* and supplementary fig. S1*N*, Supplementary Material online), 2) a calibration placed on a node subtending a rate decrease (fig. 4*C* and supplementary fig. S1*O*, Supplementary Material online), 3) both calibrations placed on nodes subtending a rate increase (fig. 4*D* and supplementary fig. S1*P*, Supplementary Material online), 4) both calibrations placed on nodes subtending a rate decrease (fig. 4*E* and supplementary fig. S1*Q*, Supplementary Material online), 5) a calibration placed on a node subtending a rate increase and a calibration placed on a node subtending a rate decrease (fig. 4*F* and supplementary fig. S1*R*, Supplementary Material online), and 6) all calibrations but one of those placed on nodes subtending a rate decrease (fig. 4*G* and supplementary S1*S*, Supplementary Material online). The application of a priori calibrations invariably improved accuracy in the Bayesian analyses. However, the improvement in divergence time estimation was not as great as in the case of the unidirectional rate changes (fig. 2). For example, the number of (uncalibrated) nodes whose generating age was within the 95% credibility intervals went from 0%, 47.2%, and 81.1% to 18.8%, 100%, and 91.7% (taking into account only the nodes that were not calibrated in either analysis) for the strict clock, uncorrelated clock, and autocorrelated clock (respectively) when the root and four nodes at 50,000 generations were calibrated. In the case of RelTime, the number of uncalibrated nodes whose generating age fell within the 95% confidence intervals went from 96.0% to 80.5%.

The strict and uncorrelated clocks continued to overestimate clade ages for fast evolving lineages and underestimate them for slowly evolving lineages, with the uncorrelated clock being more accurate in terms of mean ages and credibility intervals. The autocorrelated clock marginally underestimated the divergence time of nodes at 75,000 generations on the subtree where rate increases were applied and overestimated them in the subtree where rate decreases were implemented; this is the opposite of what was observed in the uncorrelated and strict clock. In an eight-taxon subtree where both the nodes at 50,000 generations were initially calibrated, removal of one of the two calibrations does not greatly affect the mean age estimate of the nodes descending from the ancestor that retained the calibration. When nodes whose generating age was 50,000 generations in the past were not calibrated, the divergence time estimates resemble the relative divergence time estimations shown in figure 3*A*, scaled to absolute dates (e.g., fig. 4*G*). In these cases, the accuracy of node age estimates changed from 0%, 0.8%, and 59.5% to 0%, 35.9%, and 49.0% for the strict clock, uncorrelated clock, and autocorrelated clock (respectively), averaged across all considered nodes, showing that with the exception of the uncorrelated clock, node age estimates are only improved by calibrations on (grand)parent or (grand)daughter nodes. Age estimates for these nodes under the autocorrelated clock are more accurate than when dated relatively. In RelTime, the proportion of these nodes whose generated age sits between the 95% confidence intervals went from 86.5% in the relatively dated tree to 58.6% in the a posteriori dated trees. This reflects a trend that calibrations were less effective when implemented a posteriori than a priori. This is also reflected by the positive correlation between the number of calibrations and the estimates of the extent of rate variation (sigma2 in PhyloBayes) by relaxed clock methods.

A bimodal distribution of mean age estimates for RelTime is observed when both the nodes at 50,000 generations are calibrated on the faster side of the tree, with many divergence times being overestimated. By considering each analysis individually, it is clear that the mean age estimate for a node falls under the same peak as its sister, whereas the ages of the other two nodes for which we see a bimodal distribution are under the other peak. As a result, every analysis yielded mean age estimates of two of these nodes differently from the other two, despite the branch lengths being the same (except for small differences caused by the stochasticity of the simulations). Why this is the case is unclear. When only the nodes on the slow side of the tree were calibrated, the mean age estimates of older nodes were pushed back to ∼100,000 generations as before (fig. 4*D* and *G*). The inferred relative rate for these 0-time branches was exactly 1 (as with previously discussed cases).

## Discussion

Here, we tested the power of a priori and a posteriori calibration to deconvolve the substitution rate from branch time using different methods and models. Although only a restricted set of, admittedly particularly difficult, conditions have been examined, our results show that relative divergence times and absolute divergence times obtained through a posteriori calibrated of relative divergence times are less reliable than absolute dating approaches that integrate calibrations a priori. Our simulations, designed to be especially challenging in the absence of calibrations, represent a suite of scenarios that are unlikely in nature due to the simultaneous occurrence of four independent substitution rate changes and clock-like evolution elsewhere. Although the co-occurrence of speciation events with changes in evolutionary rate as well as other speciation events is unlikely in real data sets, the magnitude of the change in evolutionary rate is reasonable when compared with some that have been described in nature (Gibbs et al. 2004; Smith and Donoghue 2008; Bromham et al. 2013; Magallon et al. 2015), although the methodology used to predict rates can strongly affect conclusions making assessment of substitution rate variation in nature difficult (see Carruthers et al. 2019). Furthermore, parallel changes in substitution rate have been inferred in empirical data (Phillips 2016; Phillips and Fruciano 2018). Our simulations are also fair in that the same conditions are applied across all methods, and differential performance under these conditions is informative of relative merits of alternative algorithms and calibration strategies. Although the number of independent substitution rate changes is larger than we would expect, our analyses show that this pattern does not need to cover the entire tree to be a problem in divergence time estimation. By not incorporating prior information on clade ages, divergence time estimates will be inaccurate on any subtree where we see independent simultaneous changes in substitution rate.

In particular, we show that under a strict clock, without prior information on clade ages, divergence times are estimated particularly poorly in the scenarios modeled. Equally, the uncorrelated clock does not find signal for the rate changes in the branch lengths but has wider credibility intervals by allowing for rate changes across branches. In relative divergence time estimation, RelTime was the most accurate method with the caveat that, in many cases, this accuracy was achieved with extreme imprecision. The autocorrelated rates model predicted divergence times slightly less accurately but with mean estimates closer to the true generating age of nodes. These results are predictable, because the way in which we modeled rate variation resembles a local clocks model (Rambaut and Bromham 1998) which autocorrelated rates are more similar to than any of the other rate variation models tested. When the relative timescales derived using RelTime were transformed into absolute timescales using a posteriori calibrations, the RelTime-estimated absolute timescales were less accurate than a priori calibrated Bayesian absolute timescales inferred under the uncorrelated and autocorrelated models (considering all node age estimates).

Adding calibrations a priori had a positive effect on all Bayesian analyses, with the greatest improvement in accuracy coming from the autocorrelated and uncorrelated relaxed clock models. In general, the more calibrations that were applied, the more accurate the inferred divergence times, in agreement with previous studies (Warnock et al. 2012; Duchene et al 2014). RelTime estimates could not be improved using calibrations as they are added a posteriori so are not used to detect rate changes. Therefore, in analyses when calibrations are available, and give important temporal information that cannot be derived from the sequences alone, Bayesian methods ought to perform better. When calibrations are not available, there are conditions where the RelTime algorithm works well. However, there are limited areas of applicability for such timescales, as it is clear that even when we say that RelTime performs well, it is only with reference to the even poorer performance of noncalibrated Bayesian methods. This is illustrated by the fact that a posteriori calibrated RelTime absolute divergence times were invariably inaccurate in these analyses, and this can only be a consequence of the fact that the RelTime relative timescale was a poor fit to start with. Our study assumed the use of correct calibrations and did not investigate the effect of incorrect calibrations on divergence time estimation. However, it is clear that although incorrect calibrations might have a negative effect on divergence time estimation, there are well-established criteria for evaluating their efficacy a priori (Benton and Donoghue 2007; Parham et al. 2012; Warnock et al. 2015; De Baets et al. 2016). According to these best practice criteria for establishing calibrations, we can ensure accuracy of calibrations at the expense of precision. Further, we propose that where possible, maximum bounds should be applied, even if the degree of precision that we can be confident with is low (see also Phillips 2016). In studies where both relative and absolute dating methods are used, and discrepancies between results are found (Eberle et al. 2018; Pie et al. 2018), we propose the results of absolute dating pipelines should be considered more reliable. We discourage the use of a posteriori conversion of relative divergence times to absolute times, as the a priori inclusion of calibration is more effective (e.g., Patane et al. 2017; Shin et al. 2018).

## Conclusions

In this study, we present simulations that violate relaxed clock models of lineage specific substitution rate variation. We show that without calibration, and in the presence of rate changes, no method tested is able to accurately infer changes in rate. These were Bayesian inference under strict clock, lognormal-autocorrelated, and uncorrelated gamma rate models as well as the local clocks-like, non-Bayesian method, RelTime. However, under our simulated conditions, RelTime and the autocorrelated clock were able to capture some of the rate variation simulated. A priori applied calibrations improve the accuracy of the estimation of node ages in a Bayesian framework. This improvement is most significant under clock models that allow for underlying substitution rate to vary by lineage: here, the uncorrelated and autocorrelated rates models. The application of calibrations a posteriori, at least as implemented in RelTime, is not helpful. If a priori implementation of calibrations is impossible, it is unclear whether any meaningful conclusions can be drawn from a molecular clock study. Overall, our results suggest that, at the least under the scenarios examined, a priori calibrated molecular clock analyses under an autocorrelated clock model is generally the most reliable method for estimating relative divergence time.

## Materials and Methods

Forward simulation of evolution was performed to generate sequences using SLiM version 2.5 (Haller and Messer 2017). Our simulation strategy ensured we could reliably calibrate phylogenies and quantify the accuracy of molecular clock methods. This was preferred to backwards, coalescent-based simulation despite increased computational burden due to the ease with which parameters such as mutation rate can be modified. Evolution was simulated assuming neutrality, with speciation events and changes in the rate of mutation at predetermined times in order to generate a phylogeny with branch lengths that require calibration in order to separate rate and time in molecular clock analyses. Modifying the substitution rate via changes in the mutation rate can be regarded as a proxy for the effects of variation in selection and population size on substitution rate (Lanfear et al. 2014). The populations were diploid, sexual, and had 500 individuals each. Populations evolved for 25,000 generations between speciation events. Speciation events were represented here by a sample of 100 individuals forming an exponentially growing population from the original population of 500. The starting sequence was taken from chromosome 2L of *Drosophila melanogaster* (Adams et al. 2000). Recombination events were simulated based on the recombination map from Comeron et al. (2012). A symmetrical tree with 16 terminals and an outgroup was used to run the simulations. Python programs were written to convert the results from SLiM to substitutions in the *Drosophila* sequence within each present-day population. Substitutions were made at positions where mutations were fixed, otherwise where the most common allele at a locus was not the ancestral allele. Where mutations occurred, each possible nucleotide change had equal probability. Each simulation was performed 100 times. The length of the sequence was 1.08 Mb. The mutation rate was $2.8\times 10^{-9}$ before any rate increases or decreases. It was $5.2\times 10^{-9}$ for fast evolving branches and $1.4\times 10^{-9}$ for slow branches. These rate variations were applied at different nodes along different branches in the tree as indicated. The complete set of rate variation schemes tested is reported in supplementary figure S1, Supplementary Material online.

Molecular clock analyses were carried out using Bayesian and non-Bayesian methods. PhyloBayes version 4.1 (Lartillot and Philippe 2004, 2006; Lartillot et al. 2007) was used for Bayesian analyses. A uniform prior on divergence times was implemented (default). The F81 substitution model was used with all rates equal to one (-poisson flag in PhyloBayes, Felsenstein 1981). The clock methods used were strict clock (-cl), lognormal-autocorrelated clock (-ln, Thorne et al. 1998), and uncorrelated gamma multipliers (-ugam, Drummond et al. 2006), implemented according to the default settings in PhyloBayes. The chain was run under a fixed tree with the correct topology using the -T option. When calibrations were implemented on the divergence times of nodes, a uniform distribution with hard upper bounds 1,000 generations older than the true date and hard lower bounds 1,000 generations younger ($U\left(49,000,\;51,000\right)$ for internal nodes and U(99,000, 101,000) for the root). No root prior was applied for uncalibrated analyses, so the age of the root was set to 1,000, as default. A rate variation applying equally to all sites was modeled. During PhyloBayes analyses, sites in the alignment that were constant across all taxa were removed, in order to reduce the amount of storage used in the chain” files. The resulting alignments (with no constant sites) were between 1,461 and 4,359, varying according to the simulation strategy and stochastic effects. Analyses ran for 10,000 cycles, sampling every cycle. The RelTime analyses were implemented using MEGACC version 7.0.26 (Kumar et al. 2016). The upper and lower bounds for divergence time calibrations were 1,000 generations either side of the true time. The HKY85 model (Hasegawa et al. 1985) was used due to the absence of an option for the F81 model in the control file for megacc. We modeled uniform rates among sites. The clock type was “local clocks.” For consistency, constant sites were also removed for RelTime analyses.

Graphics were generated in R (R Core Team 2015) using the ape package (Paradis and Schliep 2019). Data and scripts are available at the University of Bristol data repository, data.bris, at https://doi.org/10.5523/bris.uopumskkuech206ueqdxpcrif.

### Validation Tests

We tested whether our results are limited to population levels analyses. To do so, for all experiments illustrated in the Results section, we also simulated an identical tree but with 100 times the number of generations between speciation events and a 100 times lower mutation rate (supplementary figs. S2–S5, Supplementary Material online). We would expect the results from clock analyses to look identical (because the branch lengths ought to be identical) with the exception that with more generations between speciation events we would see less incomplete lineage sorting. Indeed, in all cases, the distribution of inferred dates was the same, showing our study is not limited to the population level and the findings can be applied to greater timescales.

We used different substitution models for the RelTime and Bayesian analyses. These were F81 (Felsenstein 1981) and HKY85 (Hasegawa et al. 1985). Ideally, we would have used the F81 model for both sets of analyses, but it is not available for nucleotide data in MEGA7. HKY was therefore the simplest model that could account for unequal base frequencies, despite it including a transition–transversion ratio as a free parameter. To validate this choice, we also ran the RelTime analyses under a JC69 (Jukes and Cantor 1969) model, which does not account for unequal base frequencies. The differences in results were negligible, leading us to believe that an F81 model of nucleotide substitution would give the same results of HKY in RelTime.

Convergence was investigated using tracecomp in PhyloBayes. Results were considered valid at 10,000 generations based on a trial investigation where the difference in dates between parallel runs at 10,000 and 1,000,000 cycles was negligible (on a tested subset of analyses).

The size of the alignment simulated meant that it was not feasible to run analyses in PhyloBayes without removing constant sites from the alignment. This is because the “.chain” files approach hundreds of gigabytes after a reasonable number of cycles, as well as the runtime being too long. This is not a problem in RelTime. To make sure excluding constant sites did not significantly bias our results, all RelTime analyses presented here were also analyzed without removing constant sites and the differences in node times were negligible leading to identical conclusions.

Because our simulations were completed, newer versions of MEGA were released (Kumar et al. 2018). We validated our results by running a subset of analyses in MEGAX version 10.1.2. Our results proved robust to changing the version of Mega used. One new feature of MEGAX that might be relevant to explain the appearance of 0-time branches in some of our analyses is a maximum rate ratio between branches (set to 20 by default). Despite this new parameter, branches with 0 time are inferred also using MEGAX 10.1.2. 

## Acknowledgments

A.J.B. was supported by a BBSRC SWBio-DTP PhD studentship. P.C.J.D. and D.P. by a NERC (BERT) grant (NE/P013678/1).

## Supplementary Material

evaa105_Supplementary_Data
