# Supplementary material for: Performance of A Priori and A Posteriori Calibration Strategies in Divergence Time Estimation
Source: Genome Biol Evol. 2020 May 22;12(7):1087–98. doi: 10.1093/gbe/evaa105 (PMC7486956; doi:10.1093/gbe/evaa105)
Supplement: evaa105_Supplementary_Data [file evaa105_supplementary_data.pdf]

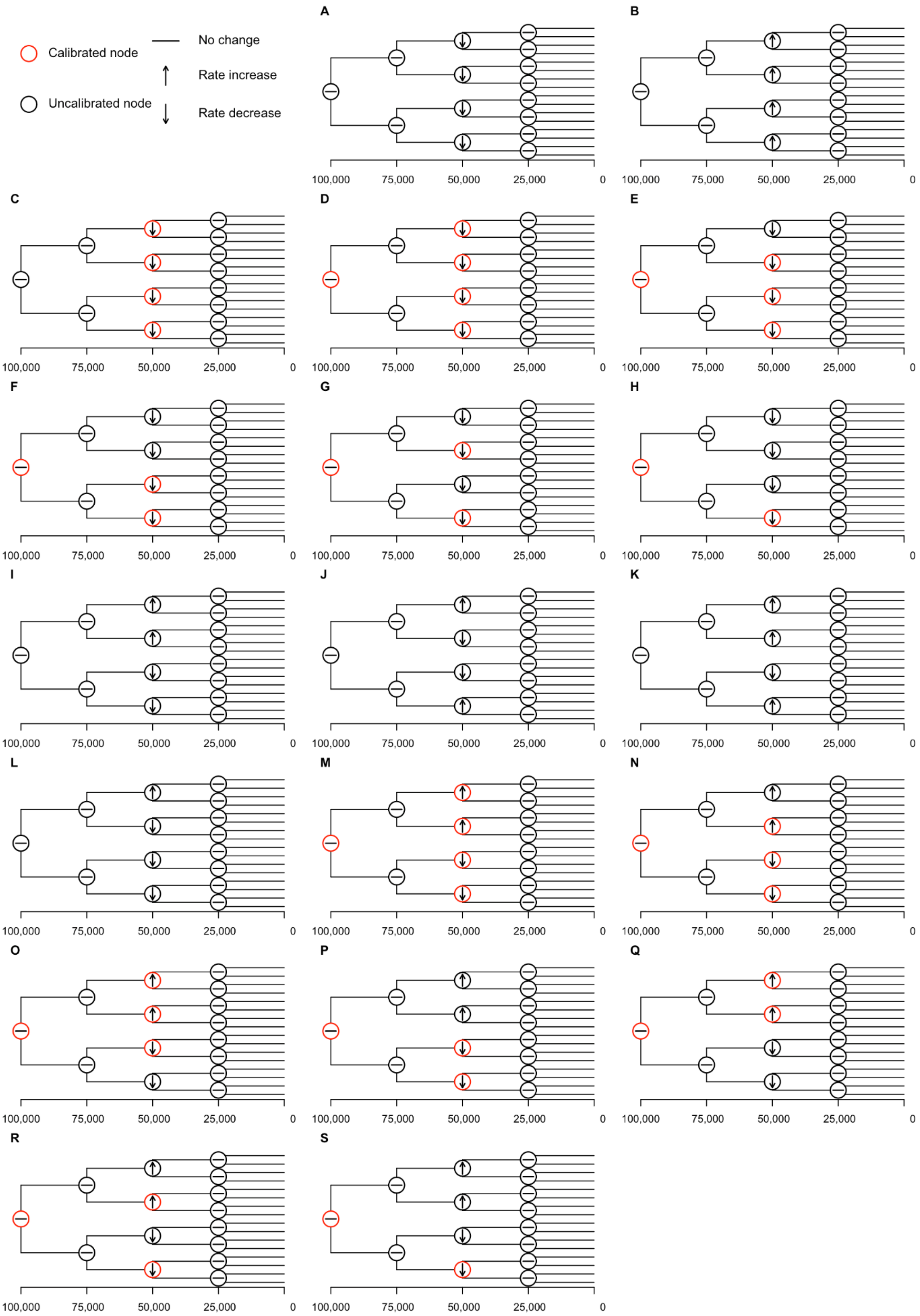

Calibrated node
Uncalibrated node
No change
Rate increase
Rate decrease
A
B
C
D
E
F
G
H
I
J
K
L
M
N
O
P
Q
R
S
100,000
75,000
50,000
25,000
0

Figure S1. The simulations and calibration strategies investigated. A key to the meaning of the symbols is present. Rate changes correspond to a 100% increase or 50% decrease, which are represented by an upwards arrow and downwards arrow respectively. Calibrated nodes are circled in red

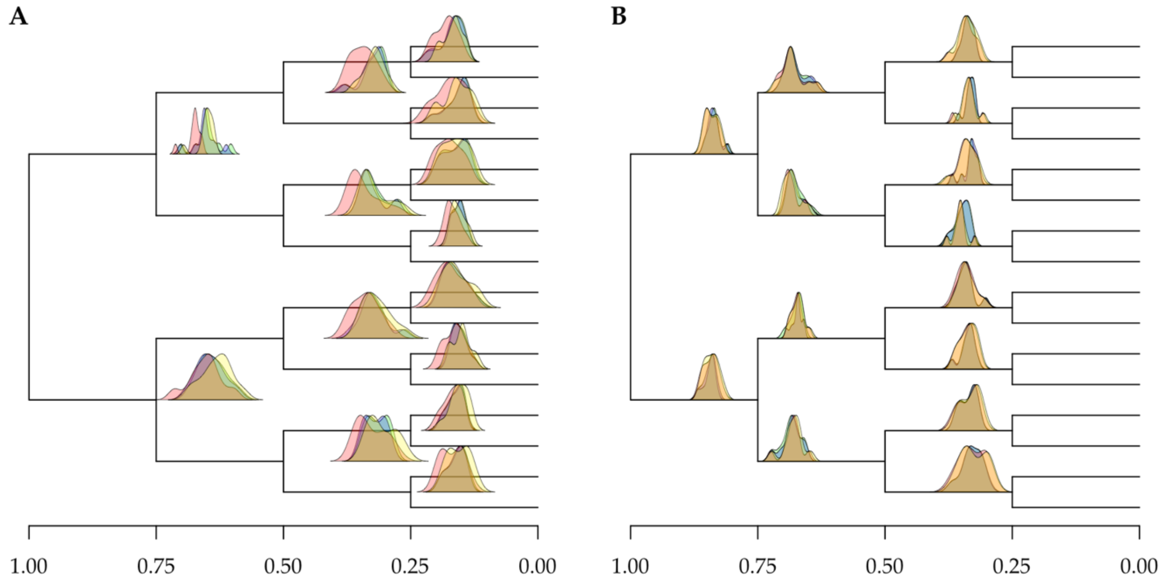

Figure S2: The distributions of node ages from a set of 10 simulations with parameters identical to those whose results are plotted in figure 1 of the main text but with a hundred-fold greater number of generations and hundred-fold lower mutation rate.

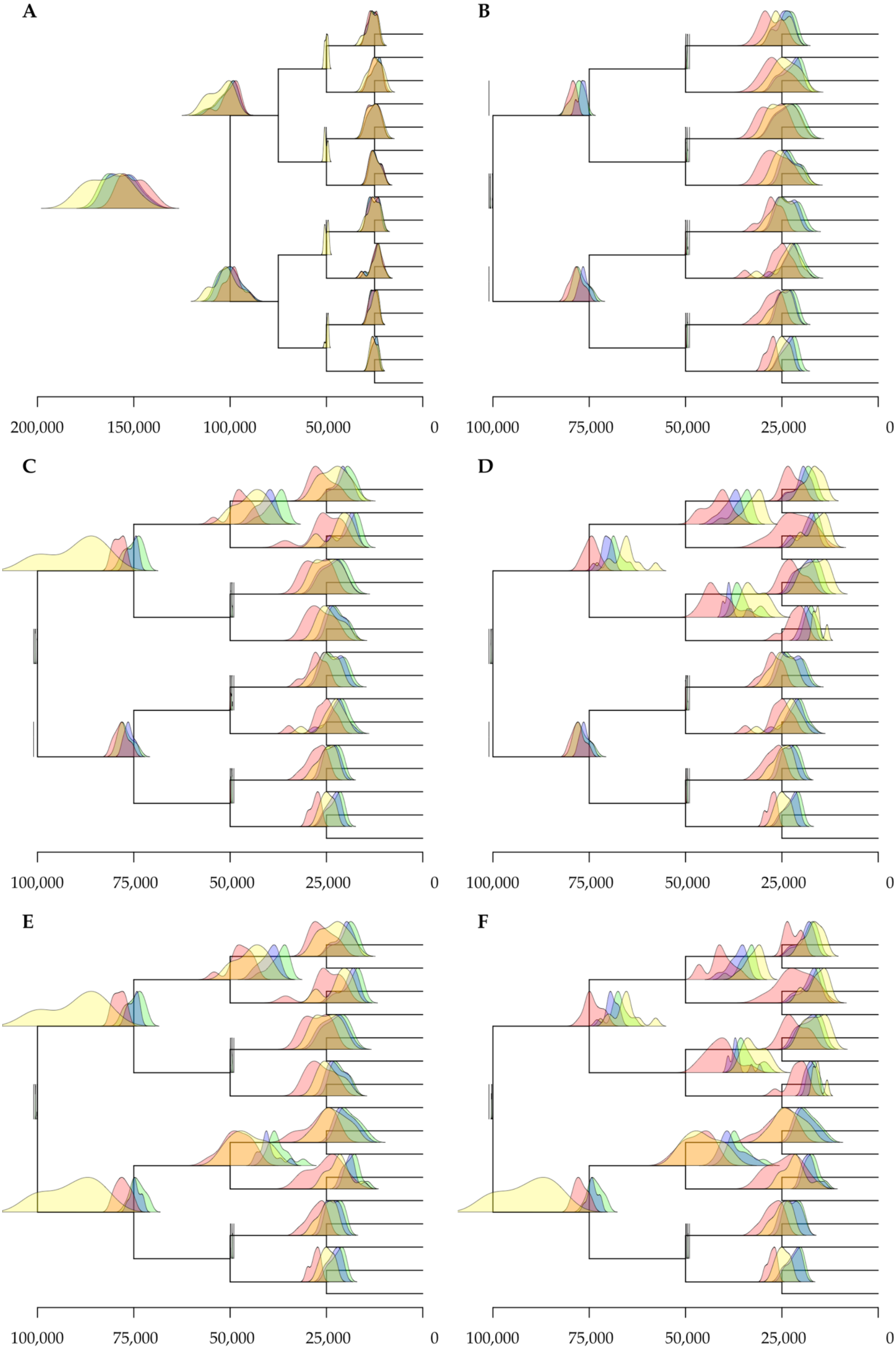
A
B
C
D
E
F
200,000
150,000
100,000
50,000
0
100,000
75,000
50,000
25,000
0

Figure S3: The distributions of node ages from a set of 10 simulations with parameters identical to those whose results are plotted in figure 2 of the main text but with a hundred-fold greater number of generations and hundred-fold lower mutation rate. The calibrations applied were the same.

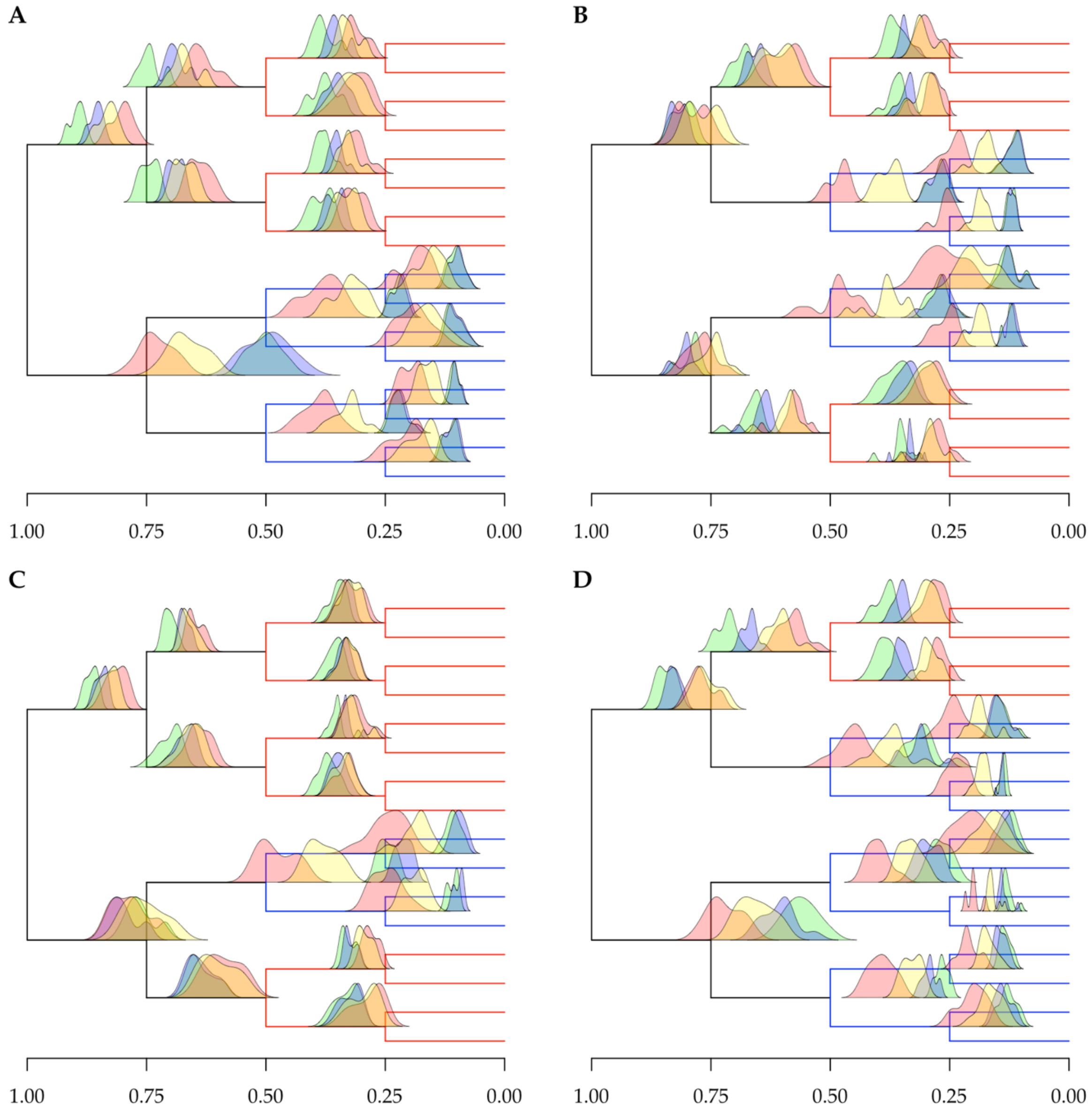

Figure S4: The distributions of node ages from a set of 10 simulations with parameters identical to those whose results are plotted in figure 3 of the main text but with a hundred-fold greater number of generations and hundred-fold lower mutation rate.

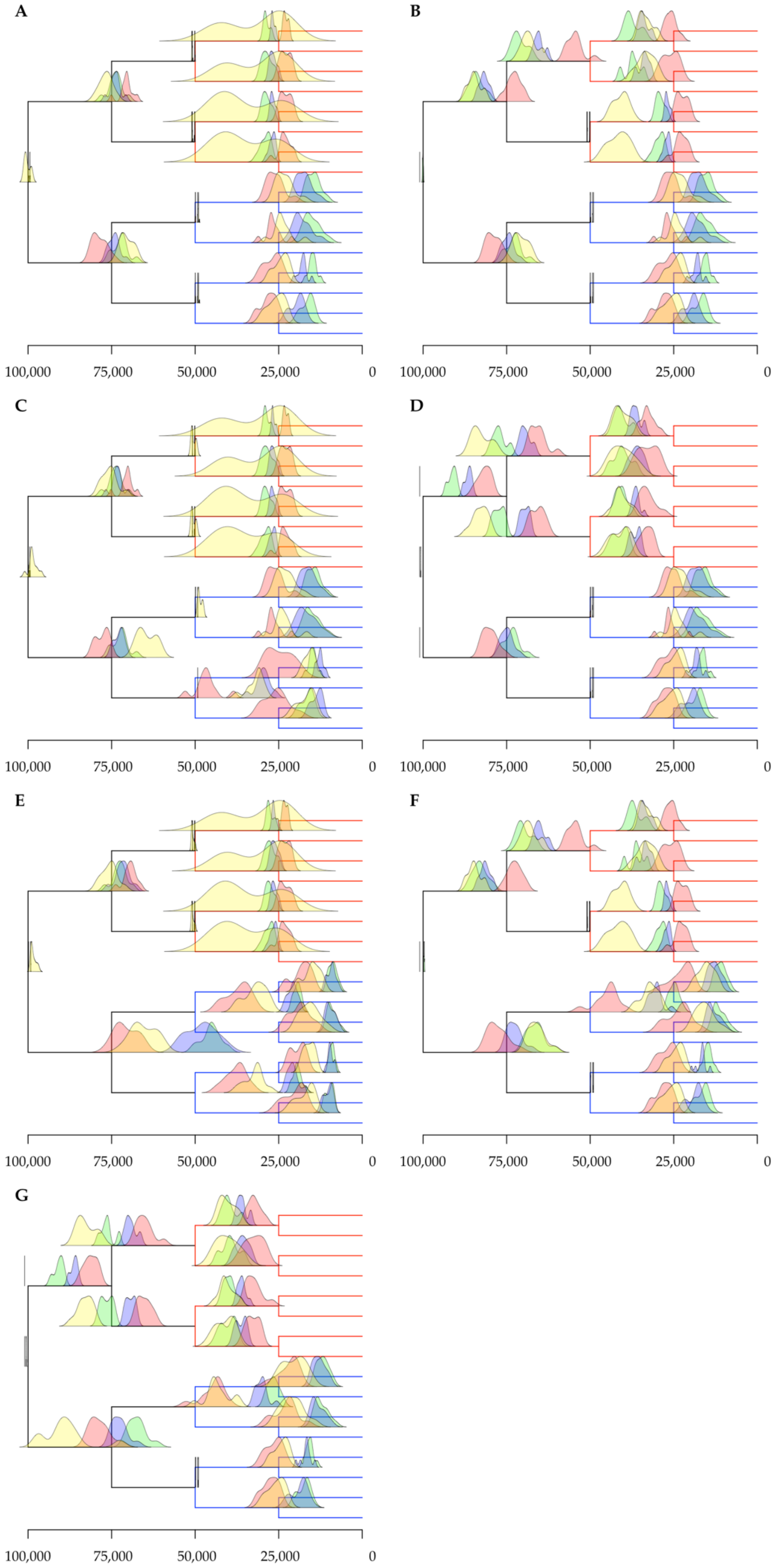
A
B
C
D
E
F
G
100,000
75,000
50,000
25,000
0

Figure S5: The distributions of node ages from a set of 10 simulations with parameters identical to those whose results are plotted in figure 4 of the main text but with a hundred-fold greater number of generations and hundred-fold lower mutation rate. The calibrations applied were the same.
